# Annotations

**Published:** 1890-07-19

**Authors:** 


### Annotations.

**The Kyrle Society.**

Those who remember the hospital wards of olden times, and who are also familiar with the wards of to-day, are able to appreciate the good work of the Kyrle Society and similar associations. The Earl of Carlisle, Sir Andrew Clarke, Mr. Burne Jones, and Mr. G. F. Watts are making a joint appeal on behalf of the Society, whose funds are not merely at a low ebb, but practically exhausted. Their appeal at the present moment is for the modest sum of £250; and this amount is wanted, not for the payment of workers, but for the purchase of materials. The Society has been in existence for thirteen years, and during that time has devoted itself to the decoration of hospital wards, parish rooms, working men's clubs, and other such places, without distinction of creed. Among those who furnish designs for the workers are such well known artists as Messrs. Cole Adams, Basil Champneys, Walter Crane, Lewis Day, Henry Holliday, James Powell, C. Harrison Townsend, and others. Whilst the names of the committee are ample guarantee of the excellence of the Society, the names of these artists furnish the best possible testimony to the value of the work done. There is no need to argue the question of the educational worth of well-designed wall decorations in hospitals, club-rooms, and other places where men, women, and children most do congregate. The cultivation of the sense of beauty is by no means one of the least important parts of education. He who can deeply admire is generally capable of much more than admiration. There were those among the Greeks of earlier and more philosophic times who held that the beautiful was identical with the good. We take a more practical, and no doubt a more correct view of moral questions than the Greeks did. But we cannot deny that the absence of the sense of beauty is often associated with low moral and intellectual endowments. On the other hand a finely developed taste helps at least the outward morals, and cannot by any means be of disservice to the heart and soul. Ugliness is hateful in itself, and cannot but be injurious. The Kyrle Society exists for the very purpose of waging war against it. We English do not flatter ourselves when we let such a society sink into pecuniary sloughs of despond. On the contrary, we rather proclaim to the world our coarseness and want of taste. Instead of supporting one such society as the Kyrle, we ought to support at least a dozen in London alone. No doubt the appeal of the Earl of Carlisle and his fellow Committeemen will be promptly responded to; but it may be hoped that those who read it will not be satisfied with giving merely temporary help to so meritorious an association, but will make it a part of their duty to furnish it with some regular provision in the future. The society could do ten times as much work as it does if it had ten times as much means, and it ought to have the means.

**Farm-Houses for Summer Holidays.**

The sun has at last condescended to visit England, and the rain has taken a very unwilling departure. Everybody has been waiting inpatiently for the change, and there will now be a universal rush to the country or the sea. Farm-houses are much sought after by a class of people whose minds are cultivated but whose purses are somewhat light. Now the ideal farm-house is idyllic as a summer holiday place. There are large and spacious sitting rooms with French windows open all day long. The bedrooms are as capacious as a London vestry hall, and the wall papers, ceilings, bed furniture, table covers, and white window curtains are as clean and fresh as soap and water and oxygen can make them. The farm-yard is a paradise for the children. The ricks and out-houses are perfect for "hide-and-seek." The hens and chickens, the ducks and geese, the cows and horses, and even the odorous pigs are unfailing sources of delight. The swing in the orchard is all but heaven itself. The pastures, the flowery banks and hedgerows, the new mown hay, the shady woods, and the quiet lanes—all these make a holiday month worthy of a fairy tale. But the ideal and the real often have no relation to each other except that of contrast. The truth is that the better class of farmers are too proud and prosperous to let their rooms to the ignorant and often conceited and patronising town-bred man. The only available places therefore are, as a rule, the houses of the smaller and less prosperous farmers. Such houses there is often great risk in occupying. The sanitary history of many a small farm-house is a very dark record. Drainage is practically unknown, except that the well from which the drinking water is drawn sometimes offers a convenient vacuum for the drippings of the farm-yard in search of a resting place. The domestic sanitation is of the most primitive and rudimentary character. It is not necessary to go into details, but if Noah was a self-respecting man, as we have reason for believing, he must have provided domestic offices at least equal to those of the average small farm-house, even after the world was denuded of its population by the flood. Notwithstanding that the ordinary farm-house is so deficient in comforts and conveniences it is much sought after, and the rent asked is correspondingly high. Still it ought not to be forgotten that those who venture into undrained and insanitary houses run a great risk. They should at least try to find out beforehand whether any infectious diseases have recently prevailed in the house or neighbourhood, and also whether the water in the well is pure, and what kind of domestic offices are available. By way of precaution they should take one or two filters with them, and filter not only the drinking water but the tea water, and also that used for cleaning the teeth. If these and other rational precautions are observed the inconveniences of a small farm-house may be endured, and much benefit may be gained by all branches of the family.

**An Open Door.**

Lady Dufferin's Fund is now being brought before the public once more. It is a great pity that there should be any lack of support. The majority of people see nothing in the movement but the providing of medical aid for Indian women. Even if the work began and ended with that, it would still be a work of rare and noble charity, and well worthy of the best aid of an intelligent and cultured community like our own. But the introduction of medical women into the households of the women of the East, and into the most intimate confidences of those women, means much more than the mere furnishing of European medical skill. It is probable that the educated English woman is as far in advance of the Indian woman intellectually as she is medically. It may be asked whether there is any advantage to themselves and the world in the intellectual advancement of Indian women? The question is supremely stupid and altogether contemptible. Yet there are many people of "weight and reputation," as Carlyle says, who ask it, and who must be answered. Here is a simple test as to whether intellectual advancement would be likely to be good for Indian women. Is the scientific medicine of Europe better for them than the absurd and useless superstitions of their own medical art? As much better as European is than native medicine so much better are the free and reasonable ideas of the West than the childish and slavish practices of the East. The education of women has been of the greatest possible advantage to this country, and promises to be more advantageous still. The bringing of a new intellect to bear upon all kinds of social, moral, and political questions is the same as the bringing of an additional sun or moon or star to shine upon a dark place. Woman's intellect, altogether different from that of man, makes visible a thousand things which man has overlooked. This is the experience of the Western Hemisphere. It need not be doubted that precisely the same experience will follow the awakening and developing of woman's intellect in the East. Lady Dufferin's movement for the supply of medical aid to Indian women has all the promise and potency of a noble intellectual revolution in it. All women who long and work for the freedom and moral advancement of their sex should seek to see the movement in its true light, and should support it both with purse and personal effort. We get a great deal done in this country that is truly humane and noble; but we do not get half as much done as we have the power to do. Our energy is not well directed; we are timid, too, and irresolute in action where we might be bold and aggressive. There is no need to think twice about such a work as Lady Dufferin has in hand. It is worthy in itself, and full of the noblest promise. What remains for the women of this country is to "go in and possess the land."

